# The calcitonin receptor protects against bone loss and excessive inflammation in collagen antibody-induced arthritis

**DOI:** 10.1016/j.isci.2021.103689

**Published:** 2021-12-24

**Authors:** Tazio Maleitzke, Alexander Hildebrandt, Tamara Dietrich, Jessika Appelt, Denise Jahn, Ellen Otto, Dario Zocholl, Anke Baranowsky, Georg N. Duda, Serafeim Tsitsilonis, Johannes Keller

**Affiliations:** 1 Charité – Universitätsmedizin Berlin, Corporate Member of Freie Universität Berlin and Humboldt-Universität zu Berlin, Center for Musculoskeletal Surgery, 13353 Berlin, Germany; 2 Berlin Institute of Health at Charité – Universitätsmedizin Berlin, Julius Wolff Institute, 13353 Berlin, Germany; 3 Berlin Institute of Health at Charité – Universitätsmedizin Berlin, BIH Biomedical Innovation Academy, BIH Charité Clinician Scientist Program, 10178 Berlin, Germany; 4 Charité – Universitätsmedizin Berlin, Corporate Member of Freie Universität Berlin and Humboldt-Universität zu Berlin, Institute of Biometry and Clinical Epidemiology, 10117 Berlin, Germany; 5 Department of Trauma and Orthopedic Surgery, University Medical Center Hamburg-Eppendorf, Martinistraße 52, 20251 Hamburg, Germany

**Keywords:** Biological sciences, Molecular biology, Immunology

## Abstract

Pharmacological application of teleost calcitonin (CT) has been shown to exert chondroprotective and anti-resorptive effects in patients with rheumatoid arthritis (RA). However, the role of endogenous CT that signals through the calcitonin receptor (CTR) remains elusive. Collagen II antibody-induced arthritis (CAIA) was stimulated in wild type (WT) and CTR-deficient (Calcr^−/−^) mice. Animals were monitored over 10 or 48 days. Joint inflammation, cartilage degradation, and bone erosions were assessed by clinical arthritis score, histology, histomorphometry, gene expression analysis, and μ-computed tomography. CAIA was accompanied by elevated systemic CT levels and CTR expression in the articular cartilage. Inflammation, cartilage degradation, and systemic bone loss were more pronounced in Calcr^−/−^ CAIA mice. Expression of various pro-inflammatory, bone resorption, and catabolic cartilage markers were exclusively increased in Calcr^−/−^ CAIA mice. Endogenous CT signaling through the mammalian CTR has the potential to protect against joint inflammation, cartilage degradation, and excessive bone remodeling in experimental RA.

## Highlights

• CT levels are increased systemically during acute experimental RA
• CTR is primarily expressed in the superficial articular cartilage layer in CAIA
• In CAIA CTR-deficiency is associated with increased inflammation marker expression
• Bone architecture is impaired in experimental RA when CTR signaling is disrupted

## Graphical abstract

**Figure:**
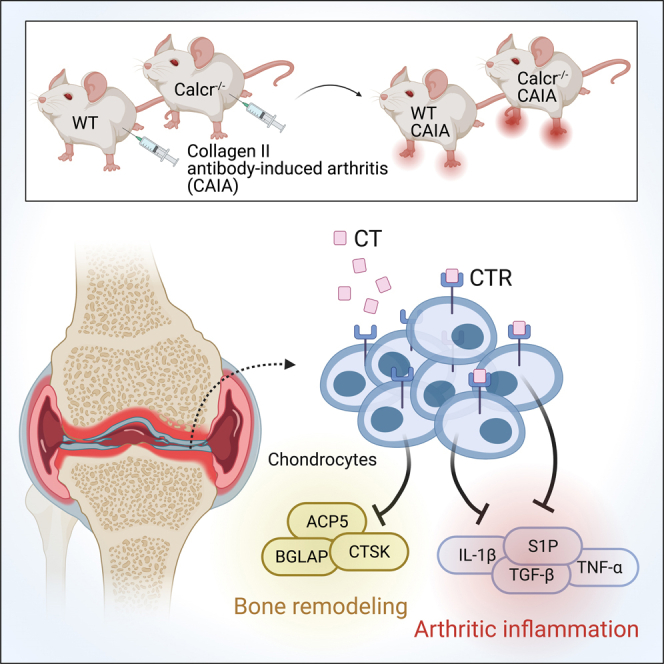

## Introduction

Rheumatoid arthritis (RA), a chronic inflammatory autoimmune disease, affects approximately 0.5% to 1% of today's population. The progressive systemic disease mainly affects symmetrical joints because of leukocyte infiltration of the synovial membrane caused by an unbalanced activation of the innate and adaptive immune system. Chondrocyte catabolism and enhanced osteoclastogenesis cause articular destruction that results in debilitating pain, joint swelling, and morning stiffness (Smolen et al., 2016).

First discovered in the 1960s, calcitonin (CT) was shown to mediate bone and cartilage turnover. Produced by parafollicular C cells of the thyroid gland, circulating CT is able to regulate osteoclast function and control the calcium and phosphate metabolism. CT binds to the calcitonin receptor (CTR), a 7-pass transmembrane protein primarily expressed in the central nervous system, the kidney, and osteoclasts. Pharmacologically employed CTR agonists, most commonly obtained from salmon or eel, exhibit a more than 50-fold higher potency than mammalian CT and have been approved by the FDA for hypercalcemic emergencies and osteoporosis treatment (Cosman et al., 2014).

Teleost CT treatment reduced systemic levels of interleukin (IL)-1 and immunoglobulins (Aida et al., 1994) and partially protected against bone erosions in patients suffering from RA (Sileghem et al., 1992). Apart from the bone sparing effects attributed to an inhibition of osteoclastogenesis and an induction of osteoblastogenesis (Karsdal et al., 2006; Sondergaard et al., 2012), treatment with teleost CT achieved a reduction of pain in murine collagen-induced arthritis (Katri et al., 2019).

After initial excitement about the broad use of teleost CT for the treatment of osteoporosis in the 1980s, rather disappointing results for fracture prevention led to an almost complete withdrawal of the costly drug from the market (Chesnut et al., 2000). In respect to RA and osteoarthritis (OA), teleost CT has not advanced into clinical application up to now (Ozoran et al., 2007).

Owing to a hitherto lack of *in vivo* evidence for a potential physiological role of CTR signaling in RA, we compared the course of collagen II antibody-induced arthritis (CAIA) in wild type (WT) and CTR-deficient (Calcr^−/−^) mice. Disease progression and resolution were assessed on functional, histological, radiological, and gene expression levels. Our results suggest a pivotal role of endogenous CT/CTR signaling with the potential to protect against excessive systemic and local inflammation during RA, in addition to preserving a physiological bone metabolism.

## Results

### Arthritis increases CT serum concentrations and the CTR is expressed in the articular cartilage of knee joints

**Figure 1:**
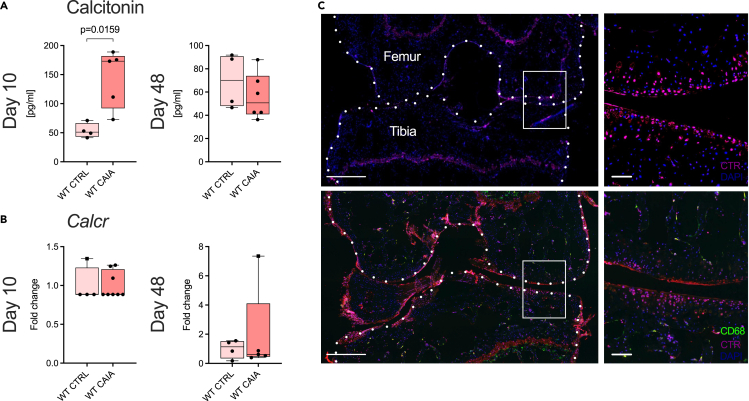
Serum CT levels are increased and the CTR is expressed in the articular cartilage during arthritis. (A) Serum CT levels in WT CTRL and WT CAIA mice on day 10 and 48. (B) Relative gene expression of *Calcr* in ankle joints derived from WT CTRL and WT CAIA mice on day 10 and 48. (C) Representative immunofluorescent stainings of coronal WT CAIA knee joint sections on day 10 using a CTR-specific antibody (purple) and blue nucleus stain (DAPI) (upper row), and an additional CD68-specific antibody (green) stain (lower row). Scale bars 500 μm (overview) and 50 μm (detail). Given values for serum CT are median ± minimum and maximum, gene expression values are median ± minimum and maximum as relative fold changes of the WT CAIA group with respect to the WT CTRL group that was set to 1.

To assess the role of endogenous CT signaling in experimental RA, we first performed serum ELISA analyses, where arthritic WT CAIA animals showed significantly higher CT levels compared to healthy controls (CTRLs) on day 10 (Figure 1A). Next, we monitored mRNA expression of *Calcr* in ankle joints of WT CAIA and CTRL mice, which remained comparatively constant over the course of arthritis (Figure 1B). Immunofluorescence of knee and ankle joints of WT CAIA mice confirmed CTR expression in corresponding superficial articular tissues, where the macrophages marker CD68 was absent (Figure 1C; Figure S1).

### CTR-deficiency does not alter clinical arthritis development but is associated with excessive acute intraarticular inflammation and concomitant cartilage degradation of knee joints

**Figure 2:**
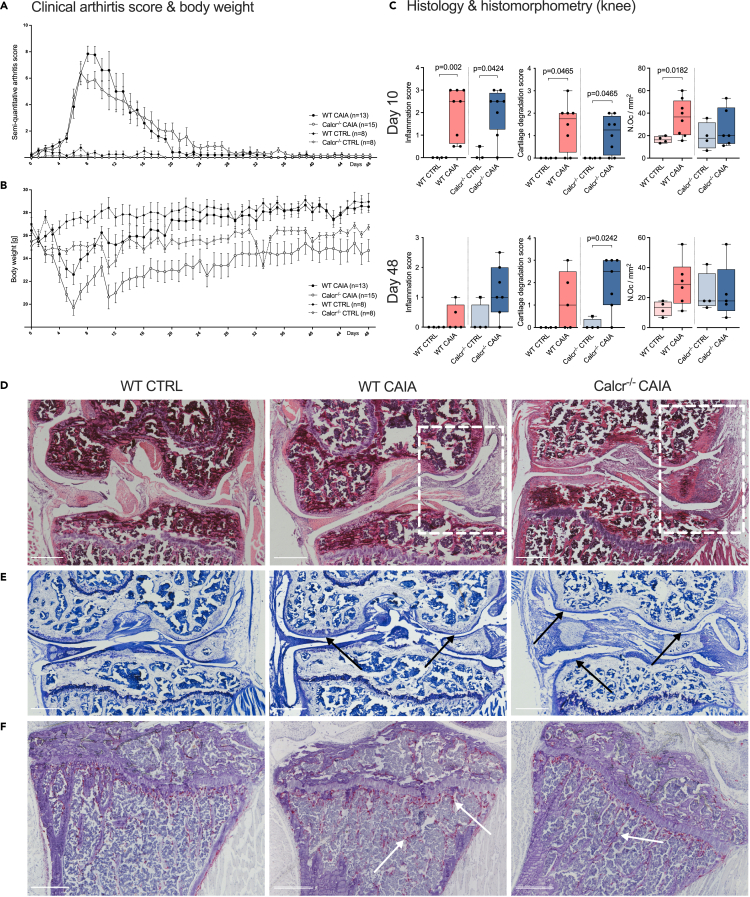
Body weight loss and cartilage degradation are more pronounced in Calcr^−/−^ animals during arthritis (A) Semi-quantitative arthritis scores and (B) body weight of indicated groups over time. (C) Histopathological inflammation and cartilage degradation scores of knee joints and number of osteoclasts of proximal tibiae on day 10 and 48. (D) Representative H&E-stained knee joint sections showing immune cell infiltration and synovial hyperplasia (white boxes), (E) toluidine-blue-stained sections showing cartilage degradation and focal defects (black arrows), and (F) TRAP-stained sections showing osteoclast zones (white arrows) on day 10. Scale bars 500 μm. Given clinical arthritis score and body weight values are mean ± SEM, given histological score values are median ± minimum and maximum.

Clinical signs of arthritis peaked around day 8 and resolved subsequently following the transient nature of the CAIA model (Figure 2A). The posterior probability of a clinical difference between WT CAIA and Calcr^−/−^ CAIA mice was 87.9% on day 8. From day 9 on, the posterior mean of WT CAIA animals dropped faster than that of Calcr^−/−^ CAIA mice, being virtually equal on day 16 (Calcr^−/−^ CAIA: 4.14 and WT CAIA: 4.18, with a posterior probability of WT CAIA having a larger score of 51.8%). From day 17 on, Calcr^−/−^ CAIA animals displayed larger estimated scores, but on a generally low level.

Body weight evaluations allow indirect assessments of overall well-being and systemic effects of arthritis. Parallel to the onset of arthritis, all animals lost body weight, before regaining weight following the clinical peak of arthritis on day 8 (Figure 2B). Between day 4 and 35, the posterior probability for Calcr^−/−^ mice to lose more weight than WT mice was consistently larger than 99%. On day 48, the posterior probability for a difference between groups was 80.4%.

In accordance with clinical findings, the histopathological inflammation score for knee joints was significantly increased in both WT CAIA and Calcr^−/−^ CAIA mice compared to CTRLs on day 10. A similar tendency was observed for Calcr^−/−^ animals on day 48 (Figures 2C and 2D). For cartilage degradation, WT CAIA and Calcr^−/−^ CAIA animals scored significantly higher than their CTRL groups on day 10. This was maintained in Calcr^−/−^ CAIA animals on day 48 while only a tendency was observed in WT mice (Figures 2C and 2E). Histomorphological assessment of proximal tibiae revealed a significantly increased number of osteoclasts on day 10 for WT CAIA mice, whereas this was not the case for Calcr^−/−^ animals (Figures 2C and 2F).

### Disrupted CTR signaling leads to pronounced inflammation and cartilage degradation in ankle joints

**Figure 3:**
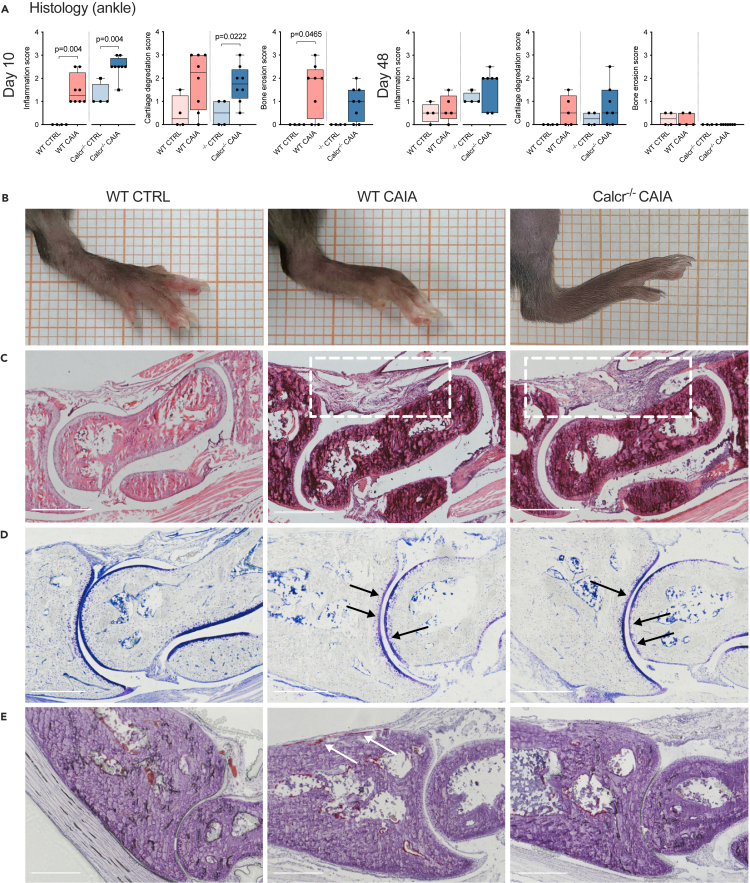
CTR-deficiency results in increased histopathological signs of arthritis (A) Histopathological inflammation, cartilage degradation, and bone erosion scores of ankle joints on day 10 and 48. (B) Clinical presentation of ankle joints on day 8. (C) Representative histopathological H&E-stained sections of ankle joints showing synovial hyperplasia, immune cell infiltration, and edema (white boxes), (D) toluidine-blue-stained sections showing cartilage degradation, indicated by loss of stain intensity (proteoglycan loss) and focal cartilage defects (black arrows) and (E) TRAP-stained sections with bone resorption zones, marked by stained osteoclast areas (white arrows) on day 10. Scale bars 500 μm. Given values are median ± minimum and maximum.

Histopathological analyses (Figure 3A) of ankle joints further confirmed clinical findings (Figure 3B), where WT CAIA and Calcr^−/−^ CAIA mice showed significantly higher inflammation scores than their respective CTRL groups on day 10. For cartilage degradation, Calcr^−/−^ CAIA mice scored significantly higher than their corresponding CTRL group on day 10, whereas only a tendency was observed in WT animals (Figures 3A and 3D). The bone erosion score on day 10 was significantly higher in WT CAIA animals compared to CTRLs, whereas this observation was much less pronounced in Calcr^−/−^ CAIA animals. As expected, no changes were observed on day 48 (Figures 3A and 3E).

### Mice lacking the CTR display impaired expression levels of cartilage and bone turnover markers

**Figure 4:**
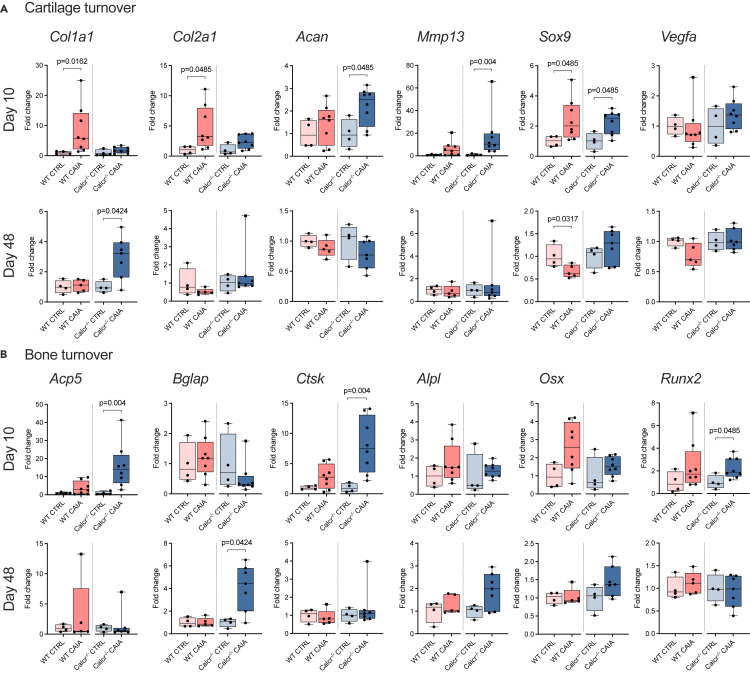
Increased expression of cartilage and bone turnover markers in CTR deficient animals (A) qRT-PCR expression analyses of cartilage turnover and (B) bone turnover markers of ankle joints on day 10 and 48. Given values are median ± minimum and maximum as relative fold changes of WT CAIA and Calcr^−/−^ CAIA groups with respect to CTRL groups that were set to 1.

To correlate histological findings with molecular data, we performed gene expression analyses of ankle joints. WT CAIA animals showed a significantly higher expression of collagen formation markers (collagen type I alpha 1 chain (*Col1a1*), collagen type II alpha 1 chain (*Col2a1*)), both crucial for repairing damaged bone and cartilage tissue (Figure 4A). In turn, only Calcr^−/−^ CAIA mice showed an increased expression of proteolytic matrix metalloproteinase 13 (*Mmp13*) and main cartilage proteoglycan, aggrecan (*Acan*), on day 10. On day 48, only *Col1a1* proved to be significantly elevated in Calcr^−/−^ CAIA mice, whereas *Sox9* was significantly reduced exclusively in WT CAIA animals.

Looking at bone turnover markers, we found an induced expression of bone resorption markers acid phosphatase 5 (*Acp5*) and cathepsin K (*Ctsk*) in Calcr^−/−^ CAIA animals on day 10, followed by an increased expression of the bone formation marker *Bglap* (encoding osteocalcin) on day 48 (Figure 4B). These data indicate that CTR-deficiency leads to an enhanced expression of bone resorption markers during acute arthritis, followed by an increase of bone formation markers during the resolution phase.

### CTR-deficiency is associated with a severe disruption of bone integrity during acute and long-term experimental RA

**Figure 5:**
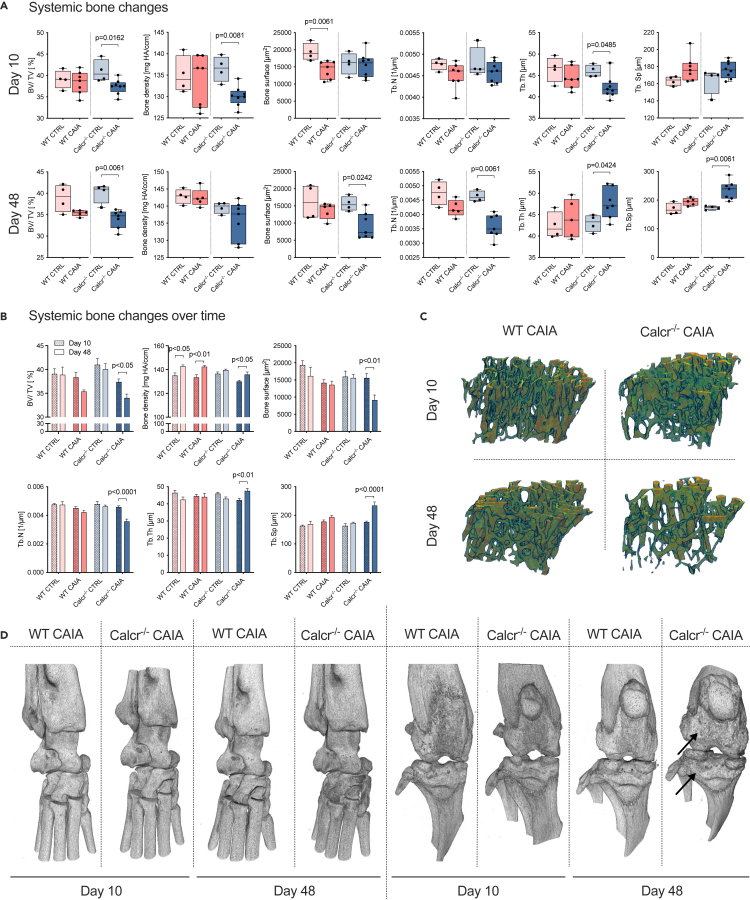
The CTR protects from systemic bone loss during arthritis (A) μCT data of systemic bone changes of proximal tibiae on day 10 and 48 and (B) of systemic bone changes over time. First columns of each group represent day 10 (dotted), second columns day 48 (filled). (C) Representative 3D μCT images of trabecular bone structure of proximal tibiae and (D) representative 3D μCT images of ankle and knee joints of WT CAIA and Calcr^−/−^ CAIA mice on day 10 vs. 48. Arrows indicate a sclerotic bone phenotype. Given values for systemic bone changes are median ± minimum and maximum, given values for systemic bone changes over time are mean ± SEM.

To further understand short-term and long-term effects of the CTR on bone remodeling during experimental RA, μ-computed tomography (μCT) analyses of proximal tibiae were conducted. On day 10, bone volume, defined as the volume of mineralized bone per total volume of interest (BV/TV), bone density, and trabecular thickness (Tb.Th) were exclusively reduced in Calcr^−/−^ CAIA mice (Figure 5A), whereas bone surface was only reduced in WT CAIA animals. On day 48, bone volume, bone surface, and trabecular number (Tb.N) were again exclusively reduced in Calcr^−/−^ CAIA mice, whereas trabecular separation (Tb.Sp) was coherently increased (Figure 5A).

To analyze systemic bone changes over time, we compared proximal tibiae on day 10 to those on day 48 within groups. For Calcr^−/−^ CAIA animals, BV/TV, bone surface, and Tb.N decreased, whereas Tb.Sp increased over time, indicating a relevant loss of bone integrity over the course of 48 days (Figures 5B–5D). Interestingly, bone density and Tb.Th increased for Calcr^−/−^ CAIA mice over time, indicating an overall loss of bone substance, with the remaining bone being remodeled to a sclerotic phenotype, most prominent in Calcr^−/−^ CAIA knee joint samples on day 48 (Figure 5D). Lastly, increased bone density was also observed for WT CAIA and WT CTRL animals, mimicking some degree of bone deposition and remodeling over time (Figures 5B and 5C).

### Inflammation and immunomodulatory costimulatory molecule expressions are predominantly increased in arthritic CTR-deficient mice

**Figure 6:**
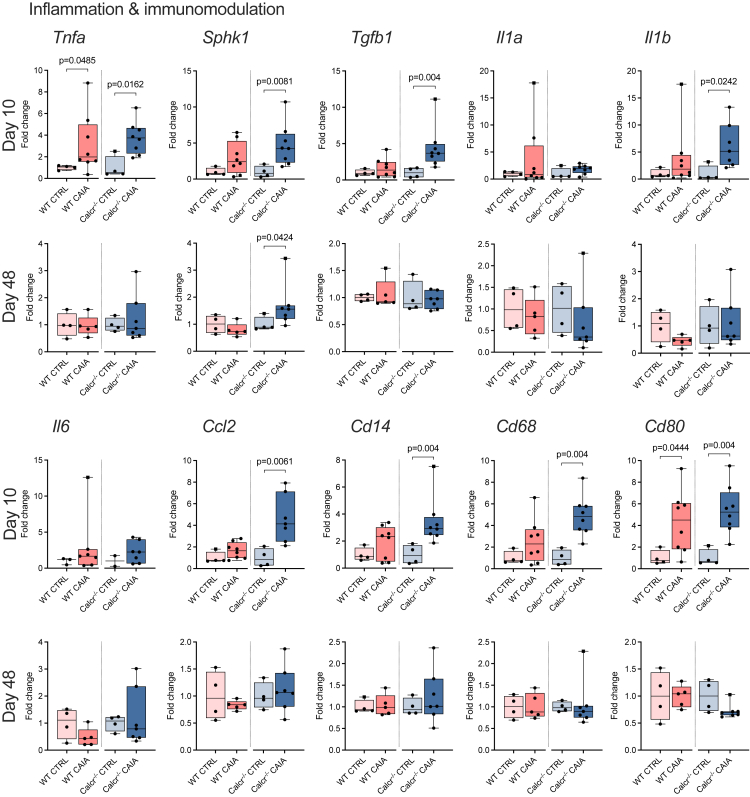
The CTR is essential to contain excessive expression of inflammation markers qRT-PCR expression analyses of inflammation and immunomodulation genes of ankle joints on day 10 and 48. Given values are median ± minimum and maximum as relative fold changes of WT CAIA and Calcr^−/−^ CAIA groups with respect to CTRL groups that were set to 1.

Finally, we conducted gene expression analyses of inflammatory markers in ankle joints. Although the expression of *Tnfa* increased in WT CAIA and Calcr^−/−^ CAIA mice, sphingosine kinase-1 (*Sphk1)*, catalyzing the conversion of sphingosine to sphingosine-1-phosphate (S1P) and thus enhancing TNF-α signaling, was only overexpressed in Calcr^−/−^ CAIA mice on day 10 and day 48 (Figure 6A). Expressions of *Tgfb*, *Il1b,* and *Ccl2* were also exclusively increased in Calcr^−/−^ CAIA animals on day 10. Corresponding to clinical results, no differences in the expression of respective genes were observed on day 48, except for *Sphk1*. Lastly, expressions of *Cd14* and *Cd68*, both macrophage surface markers, were only elevated in Calcr^−/−^ CAIA mice (Figure 6).

## Discussion

Pharmacological applications of teleost CT were previously proposed to prevent bone defects in RA (Aida et al., 1994; Ozoran et al., 2007), osteoporosis (Kaskani et al., 2005) and OA (Karsdal et al., 2015). With our current study, we unveil an independent bone and cartilage sparing, anti-inflammatory and immunosuppressive role of the mammalian CTR in antibody-mediated arthritis.

Employing the CAIA model, we were previously able to demonstrate a dual pro-inflammatory and bone protective role of the neuropeptide calcitonin gene-related peptide alpha (αCGRP) (Maleitzke et al., 2020). Although different in structure, both CT and αCGRP are encoded by the common gene *Calca* and synthesized through alternative splicing. Similar to αCGRP, the data presented herein suggest an indispensable role of the CTR in the preservation of bone integrity during inflammatory arthritis. Although αCGRP however acts as a pro-inflammatory peptide during acute experimental arthritis, we show that the CTR protects articular structures from excessive inflammation. Teleost CT was previously shown to have a high *in vivo* potency (Gydesen et al., 2016) as well as antiresorptive and chondroprotective properties in the treatment of experimental RA and OA (Karsdal et al., 2008; Sondergaard et al., 2012; Wen et al., 2016), yet little is known about the role of endogenous CT/CTR signaling in inflammatory joint diseases.

Contrary to the reported osteoprotective effects of teleost CT through an inhibition of osteoclast activity (Karsdal et al., 2008), our group could previously show that endogenous CT suppresses bone formation through the inhibition of S1P secretion from osteoclasts (Keller et al., 2014). Accordingly, our current findings indicate that CTR signaling is indispensable to control and suppress the expression of *Sphk1* during acute and chronic arthritis. *Sphk1* enzymatically generates S1P, a molecule coupling bone formation to resorption (Martin and Sims, 2015), while maintaining an inflammatory response in RA, where it is found elevated in the synovial fluid of affected patients (Hu et al., 2011; Lai et al., 2008). Inhibition of S1P led to decreased serum levels of pro-inflammatory TNF-α, IL-1β, and IL-6 and reduced arthritis activity in previous *in vivo* experiments (Lai et al., 2008; Alvarez et al., 2010), allowing the assumption that S1P is a key regulator of TNF-α stimulated IL-1β and IL-6 release in RA. Congruently, we found CTR deficient mice to display significantly elevated expression levels of *Sphk1*, *Tnfa,* and *Il1b* during acute and chronic arthritis.

Elevated TGF-β1 expression levels are found in synovial fibroblasts of RA patients and a blockade of the multifunctional cytokine has been proposed as an experimental RA treatment approach (Pohlers et al., 2007; Sakuma et al., 2007). In this study, inactivated CTR signaling led to an increased *Tgfb1* expression, underlining the anti-inflammatory effect of the CTR in antibody-mediated arthritis.

In joints, collagen synthesis is primarily dependent on *Col1a1* and *Col1a2* expression, both essential for the formation of bone and cartilage, respectively. Although WT CAIA animals showed strongly enhanced *Col1a1* and *Col1a2* expression levels during acute arthritis, this repair mechanism was not observed in Calcr^−/−^ CAIA mice. Only as arthritis subsided, *Col1a1* was increased in Calcr^−/−^ CAIA mice. Accordingly, CT treatment was previously shown to increase proteoglycan and collagen type II synthesis in human OA cartilage (Sondergaard et al., 2006, 2010). Alongside collagen synthesis marker suppression, the expression of *Mmp13* was congruently elevated in mutant animals during acute arthritis. Previous data showed intraarticular salmon CT to reduce arthritis *in vivo* and suppress the expression of MMP1, 3 and 13, all enzymes enhancing collagen fiber degradation (Ryan et al., 2013). Contradictory to our findings, CT was previously reported to enhance the expression of aggrecan (El Hajjaji et al., 2004), which can however also occur as a response to arthritic cartilage erosion and subsequent aggrecan degradation (Roughley and Mort, 2014).

In line with our previous findings regarding the function of mammalian CT as a physiological inhibitor of bone formation (Keller et al., 2014), we found the expression of the key osteoblast transcription factor *Runx2* to be significantly enhanced in Calcr^−/−^ CAIA mice, accompanied by increased expression levels of osteoclast markers *Acp5* and *Ctsk*. This indicates that during RA, the CTR is required to limit excessive bone turnover as a whole, rather than bone formation or resorption singularly. As a result, systemic bone integrity decreased markedly in Calcr^−/−^ CAIA mice over time.

Although αCGRP was previously shown to induce the production of CCL2 (Malon et al., 2011), a pro-inflammatory chemokine regulating monocyte migration in RA and OA (Raghu et al., 2017), no such data exist on CT or CTR up to now. In our study, we found that disrupted CTR signaling led to an increased expression of *Ccl2*, underlining the anti-inflammatory potential of the CTR in experimental RA. Although a first randomized controlled trial from 2006 could not show a detectable clinical benefit for CCL2 antibody therapy in RA (Haringman et al., 2006), targeting CCL2 is still being extensively discussed as a novel RA treatment approach (Moadab et al., 2021; Zhang et al., 2015; Chen et al., 2017). As CD14 (Park et al., 2016) and CD68 (Bresnihan et al., 2009) have both been proposed as biomarkers for the therapeutic response in patients with RA, our findings of increased *Cd14* and *Cd68* expression levels in Calcr^−/−^ CAIA mice, suggest a mechanistic involvement of the CTR in controlling the overexpression of these monocyte/macrophage surface markers.

The deduced functions of the CTR not only include a potent anti-inflammatory effect in affected joints, but also a protective role regarding cartilage and bone degradation during RA. Together, these results encourage further exploration of CTR signaling in RA patients, as the CTR represents a G-protein-coupled cell surface receptor known as an excellent drug target.

### Limitations of the study

There are certain limitations to this study. First, although the CAIA model exhibits several similarities to human RA, its initiation works through preformed autoantibodies mostly independent of T-cells and B-cells, which are highly relevant in human RA (Nandakumar et al., 2004). Moreover, although the murine CAIA model affects joints symmetrically similar to human RA, its transient nature is different from the chronic disease progression in affected patients. Despite these limitations, we believe that our findings are especially important from a clinical point of view, as novel water soluble CT prodrugs have recently been introduced for the treatment of osteoporosis and related musculoskeletal disorders like RA (Renawala et al., 2021).

## STAR★Methods

### Key resources table

**Table:** 

REAGENT or RESOURCE	SOURCE	IDENTIFIER
**Antibodies**		

Anti-CTR (anti-rabbit IgG, 1:300)	Bioss/BIOZOLL	Cat# bs-0124R; RRID: AB_10857260
Anti-CD68 (anti-rat IgG2a, 1:100)	Bio-Rad	Cat# MCA1957T; RRID: AB_2074849
Secondary antibody (donkey anti-rabbit IgG, 1:400)	Dianova	Cat# 711-165-152; RRID: AB_2307443
Secondary antibody (goat anti-rat IgG, 1:400, AlexaFluor 488)	Invitrogen	Cat# A-11006; RRID: AB_2534074

**Chemicals, peptides, and recombinant proteins**		

Metamizole	MSD Animal Health	Vetalgin 500mg/mL
ArthritoMab arthritis-inducing antibody cocktail	MD Bioproducts	CIA-MAB-2C
Lipopolysaccharide	MD Bioproducts	MDLPS
SCEM medium	Section-Lab Co. Ltd.	C-EM001
Trizol	Thermo Fisher Scientific	15596018
Power SYBR Green PCR master mix	Thermo Fisher Scientific	4368577
HistoReveal solution	Abcam	ab103720
Fluoromount-G	Thermo Fisher Scientific	00-4958-02
Hematoxylin and eosin stain	Merck and Chroma - Waldeck	109253 and 2C-140
Toluidine-blue stain	Sigma-Aldrich	T3260
Sodium accetate	Merck	106268
Sodium tartrate dihydrate	Merck	106663
Dimethylformamide	Sigma-Aldrich	D4551
Triton X-100	Sigma-Aldrich	T8787
Naphtol AS-MX-phosphate	Sigma-Aldrich	N5000
Fast Red Violett LB salt	Sigma-Aldrich	F3381
Mayer’s Hemalum solution	Merck	109249

**Critical commercial assays**		

RNeasy mini	Qiagen	74106
RevertAid First Strand cDNA synthesis kit	Thermo Fisher Scientific	K1621
Mouse Calcitonin (sandwich ELISA) kit	LSBio	LS-F23047

**Experimental models: Organisms/strains**		

Mus musculus: Wild type (C57Bl/6J)	The Jackson Laboratory	000664
Mus musculus: Calcr^-/-^ (C57Bl/6J)	Emeson (Vanderbilt University)/Amling (University of Hamburg) laboratories	MGI: 5751436

**Oligonucleotides**		

Primers for qRT-PCR	Eurofins Genomics GmbH	See STAR Methods

**Software and algorithms**		

AxioVision40 v4.8.2.0	Carl Zeiss	N/A
Fiji image processing package	ImageJ version 1.53e	N/A
DATAVIEWER v1.5.2.4	Bruker	N/A
3D.SUITE/CTVOX v3.3.0r1403	Bruker	N/A
Sequence Detection System v2.4	Thermo Fisher Scientific	4350490
Reference Genome GRCm38.p6 C57BL/6J	N/A	N/A
Primer3 software v.0.4.0	Whitehead Institute for Biomedical Research	http://bioinfo.ut.ee/primer3-0.4.0/
brms package	R	(Bürkner, 2017)

**Other**		

CM3050 S Kryostat cryo microtome	Leica Biosystems	14903050S01
Zeiss Axioskop 40	Carl Zeiss	N/A
SkyScan 1172 scanner	Bruker	10K01158
Ultra Turrax disperser T25 D	IKA Werke	0003725000
NanoPhotometer P360	Implen GmbH	6036
7900HT Fast Real-Time PCR system	Thermo Fisher Scientific	4329001
All-in-One fluorescence microscope BZ-X810	Keyence	8D810026

### Resource availability

#### Lead contact

Further information and requests for resources and reagents should be directed to and will be fulfilled by the lead contact, Johannes Keller (j.keller@uke.de).

#### Materials availability

This study did not generate new unique reagents.

#### Data and code availability

- Data: All data reported in this paper will be shared by the lead contact upon request.
- Code: This paper does not report original code.
- Additional information: Any additional information required to reanalyze the data reported in this paper is also available from the lead contact upon request.

### Experimental model and subject details

#### Animals

In the global CTR deficiency model (Calcr^−/−^), exon 6 and 7 of the *Calcr* gene are excised, rendering the receptor protein dysfunctional (Keller et al., 2014). All mice were backcrossed into a pure C57Bl/6J genetic background at least seven times and kept at a 12h light/12h dark cycle, fed a standard diet with access to water ad libitum, enriched with analgetic metamizole. Group housing was ensured.

In accordance with 3^R^ principles, WT animals served as a positive CTRL group in a previous experiment, conducted six weeks prior to the present one, following the same standardized protocol (Maleitzke et al., 2020). Animal allocation to experimental groups was based on simple randomization for each genotype. Ethical approval (G-0044/18) was obtained from the local animal welfare organization (Landesamt für Gesundheit und Soziales in Berlin, Germany) and all experiments were carried out in accordance with institution guidelines.

### Method details

#### CAIA model

Intraperitoneal (i.p.) injections of 8 mg of ArthritoMab arthritis-inducing antibody cocktail (20 mg/mL) and 100 μg of lipopolysaccharide (both MD Bioproducts, Oakdale, MN, USA) were conducted on day 0 and 3 to facilitate CAIA development in 15 Calcr^−/−^ CAIA and 13 WT CAIA mice. WT CTRL (n = 8) and Calcr^−/−^ CTRL mice (n = 8) received respective i.p. injections of sterile phosphate buffered saline (PBS). The ArthritoMab antibody cocktail is based on monoclonal antibodies to collagen type II, causing a complement-dependent inflammatory joint reaction (Nandakumar et al., 2003). The CAIA model is sex- and age-dependent, with male and aged animals being more susceptible to CAIA. A protective role of estradiol against CAIA is being discussed (Nandakumar et al., 2003). To ensure arthritis onset and progression, male mice aged 10-12 weeks were chosen for all experiments.

#### Study design

All animals were monitored daily over the course of 10 or 48 days. To assess the acute inflammatory phase of arthritis, 8 Calcr^−/−^ CAIA and 8 WT CAIA animals in addition to 4 Calcr ^−/−^ CTRL and 4 WT CTRL animals were euthanized on day 10. To investigate the chronic or resolution phase of arthritis, 7 Calcr^−/−^ CAIA and 5 WT CAIA, 4 Calcr^−/−^ CTRL and 4 WT CTRL mice were euthanized on day 48.

#### Arthritis score, weight, humane endpoints

Arthritis was assessed daily, following weighing of animals, employing a semiquantitative clinical arthritis score (Lee et al., 2006) by two blinded observers (T.M. and A.H.). A score between 0 and 3 was given based on redness, swelling and number of affected digits for each paw and scores were added up (maximum score of 12).

0: No swelling; 1: Mild to moderate swelling and erythematic ankle and/or 1 swollen digit; 2: Moderate swelling and erythematic ankle or swelling in 2 or more digits; 3: Marked swelling along all aspects of the paw or all 5 digits swollen.

Weight loss of >30% compared to baseline without recovery within 24h, limping and avoidance of movement and grooming were defined as humane endpoints. The clinical arthritis score served as the primary experimental outcome. All other parameters served as secondary outcomes.

#### Sample preparation

Left ankle joints were carefully dissected, stripped of all muscle and soft tissue and snap frozen in liquid nitrogen for RNA isolation and gene expression analysis. Right ankle and knee joints were fixed in paraformaldehyde (PFA) 4% before μCT analysis. After radiologic analysis, samples were embedded in SCEM medium as previously described (Kawamoto and Shimizu, 2000).

#### Histology

Embedded samples were stored at −80°C and serial sections of 7 μm were cut using a CM3050 S Kryostat cryo microtome (Leica Biosystems, Wetzlar, Germany). Central coronal views of knee joints and central lateral views of ankle joints were obtained and sections were stained with 1) hematoxylin and eosin (H&E), 2) toluidine-blue and 3) tartrate-resistant acid phosphatase (TRAP). A previously established scoring system from 0 to 3 was applied by two blinded investigators (T.M. and A.H.) (Tu et al., 2016; Bendele et al., 1999).

*H&E:* Inflammation score: 0: normal; 1: mild inflammatory infiltration with no soft tissue edema or synovial lining cell hyperplasia; 2: moderate infiltration with surrounding soft tissue edema and some synovial lining cell hyperplasia; 3: severe infiltration with marked soft tissue edema and synovial lining cell hyperplasia.

*Toluidine-blue:* Cartilage score: 0: normal; 1: mild loss of toluidine-blue-staining; 2: moderate loss of toluidine-blue-staining and cartilage loss; 3: marked loss of toluidine-blue-staining with marked multifocal cartilage loss.

*TRAP:* Bone erosion score: 0: normal; 1: mild (some areas of resorption not readily apparent on low magnification with visible osteoclasts); 2: moderate (obvious bone resorption with a few osteoclasts visible); 3: marked (large erosion areas extending into the bone cortex with numerous osteoclasts visible in all areas).

#### Histomorphometry

TRAP-stained proximal tibial sections were used to morphologically identify and quantify osteoclasts. A Zeiss Axioskop 40 (Carl Zeiss, Oberkochen, Germany) was used in combination with AxioVision40 v4.8.2.0 (Carl Zeiss) at a magnification of 40. A region of interest consisting of five square fields (300 μm × 300 μm) was set 300 μm distally of the center of the tibial growth plate within the trabecular bone.

#### μCT

A SkyScan 1172 scanner (Bruker, Billerica, MA, USA) was used to acquire μCT scans of knee joints at 70 kV and 142 μA with a slice thickness of 5.1 μm. Systemic bone changes were evaluated in proximal tibiae where a volume of interest (VOI) of 1 mm in length was placed around the outer cortical bone layer, starting 0.5 mm below the most distal point of the growth plate. Assessed global bone parameters included BV/TV in % and bone density in mg Hydroxyapatite (HA)/ccm, as well as trabecular bone parameters, including bone surface in μm^2^, Tb.N in 1/μm, Tb.Sp in μm and Tb.Th in μm. NRecon reconstruction software (SkyScan, Bruker) was used for section reconstruction. To quantify the trabecular and cortical morphometry of the bone, the Fiji image processing package of ImageJ was used. For 3D image reconstruction we used DATAVIEWER (Bruker) and 3D.SUITE/CTVOX (Bruker) software.

#### Quantitative real time polymerase chain reaction (qRT-PCR)

Snap-frozen ankle joint samples were trimmed to their corresponding tibiotalar articular surfaces. This resulted in samples including cartilage, small amounts of synovium, periarticular tissue, and minimal bone tissue. Samples were homogenized using an Ultra Turrax disperser (IKA-Werke, Staufen, Germany) with Trizol (Thermo Fisher Scientific, Waltham, MA, USA). RNA was isolated with the RNeasy mini Kit (Qiagen, Hilden, Germany), including DNase I treatment. Concentrations were determined and purity monitored (A260/A280 ratio of 1.9–2.1) using a NanoPhotometer P360 (Implen GmbH, Munich, Germany). 0.25–1 μg of RNA was reverse transcribed to complementary DNA (cDNA) using the RevertAid First Strand cDNA Synthesis Kit (Thermo Fisher Scientific). qRT-PCR was performed on 384 well-plates in a 7900HT Fast Real-Time PCR System (Thermo Fisher Scientific), employing Sequence Detection System (SDS) software (Thermo Fisher Scientific). The Power SYBR Green PCR master mix (Thermo Fisher Scientific) was run at an annealing temperature of 60°C with 2 ng of cDNA per well. For each run threshold cycle (Ct), and melting curve were calculated, confirming PCR product specificity.

Primers were individually designed using the GRCm38.p6 C57BL/6J reference genome and employing Primer3 software (http://bioinfo.ut.ee/primer3-0.4.0/), spanning at least two exons with a large intron in between to avoid genomic DNA amplifications. Primers were obtained from Eurofins Genomics GmbH (Luxemburg) and used at a final concentration of 0.2 μM. Gene expressions were normalized per housekeeping gene, glycerinaldehyde-3-phosphate-dehydrogenase (*Gapdh*), and values for WT CAIA and Calcr^−/−^ CAIA groups are displayed as fold changes relative to respective CTRL samples.

Primer sequences: *Acan* forward CAATTACCAGCTGCCCTTCA, *Acan* reverse CAGGGAGCTGATCTCGTAGC, *Acp5* forward GGTATGTGCTGGCTGGAAAC, *Acp5* reverse ATTTTGAAGCGCAAACGGTA, *Alpl* forward GCACCTGCCTTACCAACTCT, *Alpl* reverse TTTGGAGTTTCAGGGCATTT, *Bglap* forward CCTGGCTGCGCTCTGTCT, *Bglap* reverse TGCTTGGACATGAAGGCTTTG, Calcr forward TGGGTCACTCCTTGTCGATT, *Calcr* reverse CTTGTGCAAGGTCACCCTCT*, Ccl2* forward AGCTGTAGTTTTTGTCACCAAGC, *Ccl2* reverse GACCTTAGGGCAGAT, *CD14* forward CCCAGTCAGCTAAACTCGCT, *Cd14* reverse AGGGTTCCTATCCAGCCTGT, *Cd68* forward ACACTTCGGGCCATGTTTCT, *CD68* reverse GGGGCTGGTAGGTTGATTGT, *Cd80* forward CAAGTTTCCATGTCCAAGGC, *Cd80* reverse GGCAAGGCAGCAATACCTTA, *Col1a1* forward TGTTCAGCTTTGTGGACCTC, *Col1a1* reverse TCAAGCATACCTCGGGTTTC, *Col2a1* forward GGTCCCCCTGGCCTTAGT, *Col2a1* reverse CCTTGCATGACTCCCATCTG, *Ctsk* forward GTCGTGGAGGCGGCTATATG, *Ctsk* reverse AGAGTCAATGCCTCCGTTCTG, *Gapdh* forward ACTGAGCAAGAGAGGCCCTA, *Gapdh* reverse TATGGGGGTCTGGGATGGAA, *Il1a* forward TCGCAGCAGGGTTTTCTAGG, *Il1a* reverse TGCAGGAATGTACGGAGAGC*, Il1b* forward ACCTAGCTGTCAACGTGTGG, *Il1b* reverse TCAAAGCAATGTGCTGGTGC, *Il6* forward CCCCAATTTCCAATGCTCTCC, *Il6* reverse CGCACTAGGTTTGCCGAGTA, *Mmp13* forward GATGGCACTGCTGACATCAT, *Mmp13* reverse TTGGTCCAGGAGGAAAAGC, *Runx2* forward GTGGCCACTTACCACAGAGC, *Runx2* reverse TGAGGCGATCAGAGAACAAA, *Sox9* forward AAGACTCTGGGCAAGCTCTG, *Sox9* reverse GGGCTGGTACTTGTAATCGGG, *Sphk1* forward AGGTGGGGCTATGACTTGGA, *Sphk1* reverse CCCAGGGAAGGTCCCTAAGA, *Sp7 (Osx)* forward GCCCCCTGGTGTTCTTCATT, *Sp7 (Osx)* reverse CCCATTGGACTTCCCCCTTC, *Tgfb1* forward GCTGGCCTCGATACTCATCT, *Tgfb1* reverse CTTCATCTGGGCGTCGAT, *Tnfa* forward ACGCTGATTTGGTGACCAGG, *Tnfa* reverse GACCCGTAGGGCGATTACAG, *Vegfa* forward TCTCCCAGATCGGTGACAGT, and *Vegfa* reverse AAGGAATGTGTGGTGGGGAC.

#### Fluorescent immunohistochemical staining

Frozen sections were thawed, washed with PBS, treated with HistoReveal solution (ab103720, Abcam, Cambridge, UK) and blocked with 7% bovine serum albumin (BSA)/PBS. Anti-CTR (anti-rabbit IgG, 1:300, Bioss/BIOZOLL, Cat# bs-0124R, RRID:AB_10857260) and anti-CD68 (anti-rat IgG2a, 1:100, Bio-Rad, Cat# MCA1957T, RRID:AB_2074849) incubation overnight was followed by secondary antibody (donkey anti-rabbit IgG, 1:400, Dianova, Cat# 711-165-152, RRID: AB_2307443; goat anti-rat IgG, 1:400, AlexaFluor 488, Invitrogen, Cat# A-11006, RRID:AB_2534074) incubation for 1h and Fluoromount-G mounting with DAPI (Thermo Fisher Scientific). Images were acquired using an inverted fluorescence phase contrast microscope (Keyence, Osaka, Japan).

#### Biochemical assays

Serum CT concentrations were determined using an enzyme-linked immunosorbent assay (ELISA) kit according to the manufacturer's instructions (LSBio LS-F23047).

#### Sample size

We aimed for group sizes of 8 animals with an allocation ratio of 2:1 in favor of CAIA compared to CTRL animals. This sample size allows to detect large effect sizes of Cohen's d = 2.0 with 80% power and a two-sided type I error rate of 5%, when comparing CAIA vs. CTRL with a t test for independent groups. For comparisons between two CAIA groups, effect sizes of d = 1.5 could be detected. However, statistical power of the analyses of relevant outcomes was considerably higher since for each subject, multiple time points of observation (up to 48) were planned. Appropriate statistical models that account for repeated measurements guaranteed a much larger statistical power, so that also smaller effect sizes could be observed.

### Quantification and statistical analysis

Bayesian ordinal mixed effects regression models were implemented with a random intercept for individual mice to assess clinical score and body weight where values were standardized as a percentage of individual baseline measurements. The Bayesian framework allows fitting of complex models and provides an intuitive interpretation of estimates in terms of posterior probabilities, instead of p-values and confidence intervals. As explaining variables, genotype (WT or Calcr^−/-^), CAIA (yes or no) and time (in days) were applied. To account for non-linearity, a variable indicating that animals were in an arthritis initiation phase from day 0 to 7 was used. Models were fitted using the brms package in R (Bürkner, 2017).

Endpoint comparisons between groups were performed using the Wilcoxon-Mann-Whitney-test*.* To assess bone changes over time, a 2-way ANOVA analysis of variance with multiple comparisons was used. To detect outlier values, the Grubb's Test was performed for each group and outliers were displayed as square data points, while remaining data points appear as circles. Outliers were included in all analyses. Unless stated otherwise, data are presented as mean ± standard error of the mean (SEM) or median ± minimum and maximum. Significance was accepted where p <0.05. Based on the pronounced phenotypical differences between WT and CTR-deficient animals already at baseline (i.e., unchallenged), statistical differences were calculated based on relative changes induced by CAIA within mice of each genotype. For data reporting and storage, we followed the internationally established ARRIVE guidelines (Kilkenny et al., 2010). The graphical abstract was created using BioRender.com.
