# Supplementary material for: The calcitonin receptor protects against bone loss and excessive inflammation in collagen antibody-induced arthritis
Source: iScience. 2021 Dec 24;25(1):103689. doi: 10.1016/j.isci.2021.103689 (PMC8753130; doi:10.1016/j.isci.2021.103689)
Supplement: Document S1. Figure S1 [file mmc1.pdf]

iScience, Volume *25*

Supplemental information

The calcitonin receptor protects against bone loss and excessive inflammation in collagen antibody-induced arthritis

Tazio Maleitzke, Alexander Hildebrandt, Tamara Dietrich, Jessika Appelt, Denise Jahn, Ellen Otto, Dario Zocholl, Anke Baranowsky, Georg N. Duda, Serafeim Tsitsilonis, and Johannes Keller

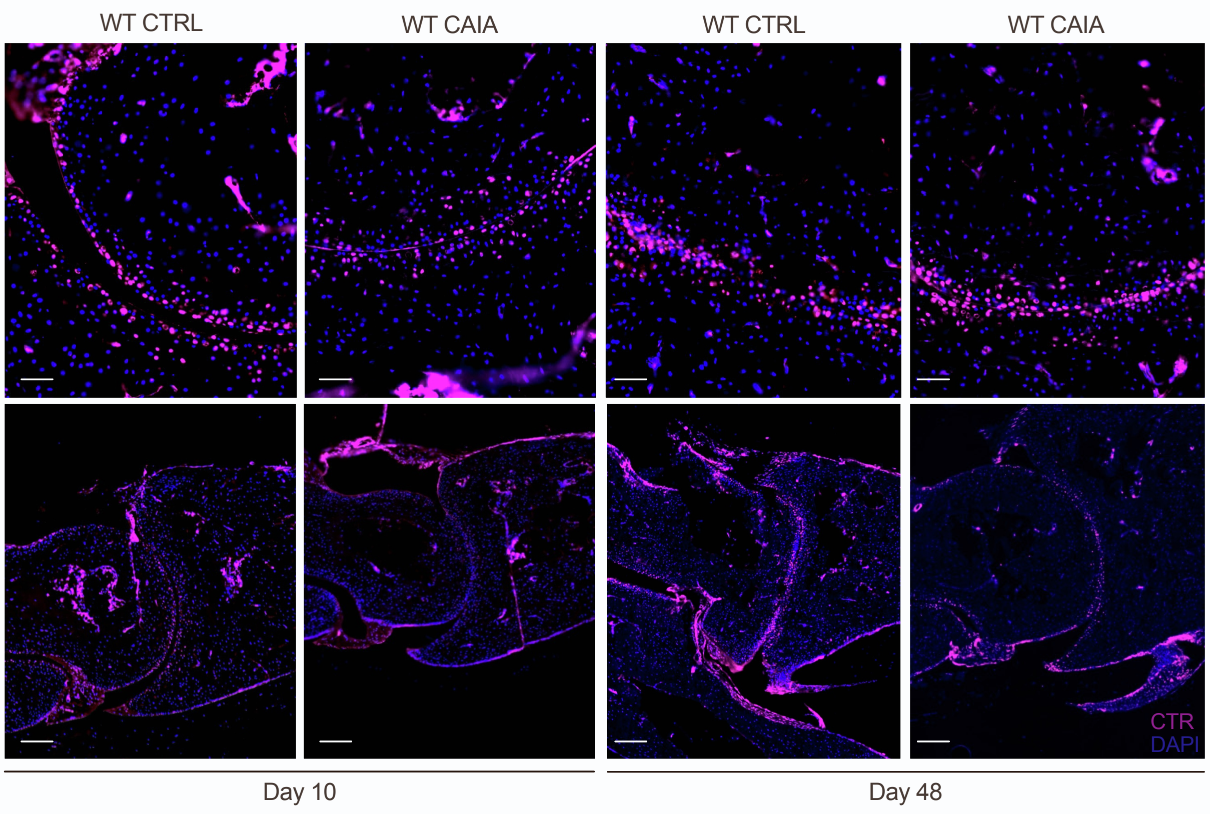

Figure S1. CTR distribution in WT CTRL and WT CAIA ankle joints, related to Figure 1. Representative immunofluorescent stainings of sagittal ankle joint sections on day 10 and 48 using a CTR-specific antibody (purple) and blue nucleus stain (DAPI). Scale bars 50 µm (upper row) and 500 µm (lower row).
